# Patient passports for rare diseases: results of a pilot study

**DOI:** 10.1038/s41431-025-01930-w

**Published:** 2025-10-14

**Authors:** Jo Balfour, Vaila Morrison, Lydia Seed, Joseph Clymer, Emma Warnants, Abigail Lampkin, Sarah M. Leiter, Gemma Chandratillake

**Affiliations:** 1 Cambridge Rare Disease Network, Cambridge, UK; 2 Department of Public Health and Primary Care, University of Cambridge, Cambridge, UK; 3 George Elliot Hospital, Coventry, UK; 4 Costello Medical, London, UK; 5 Cambridge University Hospitals, Cambridge, UK; 6 Department of Paediatrics, University of Cambridge, Cambridge, UK; 7 NHS East Genomics, Cambridge, UK; 8 Department of Genomic Medicine, University of Cambridge, Cambridge, UK

**Keywords:** Health care, Quality of life

## Abstract

For individuals with rare diseases, complex needs requiring multidisciplinary management can cause disjointed healthcare and challenges communicating with healthcare professionals (HCPs). ‘Patient passports’ support communication and healthcare coordination by sharing healthcare information with HCPs, reducing burden on patients/caregivers. Currently, no widely adopted passport addresses the multifaceted needs of patients with rare diseases. This pilot study was a service evaluation of a rare-disease-specific patient passport, co-designed with patients and HCPs. Patients/caregivers completed surveys before (‘pre-passport’) and after (‘post-passport’) using the passport. HCPs were surveyed on their perception of the passport. Of 157 ‘pre-passport’ survey respondents, 96.2% spent considerable time explaining medical needs to new care teams; 65.6% found communicating care needs challenging. Nearly all respondents (99.4%) believed a document presenting relevant healthcare information would be helpful. Among 55 ‘post-passport’ survey respondents, 85.1% used the passport during care interactions; 72.2% found it eased communication with unfamiliar teams, and 64.2% felt more confident communicating their needs. Over half (53.8%) felt the passport helped access needed care, 67.9% found it more useful than existing tools, and 75.9% were highly likely to recommend it to peers. All 31 HCP respondents listed perceived benefits, including improved HCP-patient/caregiver communication; some noted a preference for formal endorsement. By alleviating patient/caregiver-HCP communication challenges, this rare-disease-specific patient passport can enhance healthcare coordination and patient experiences. Participants’ use of the passport during interactions with care teams and likelihood of recommendation to peers support its widespread integration. Further work to assess usability across healthcare settings and to gain formal endorsement is warranted.

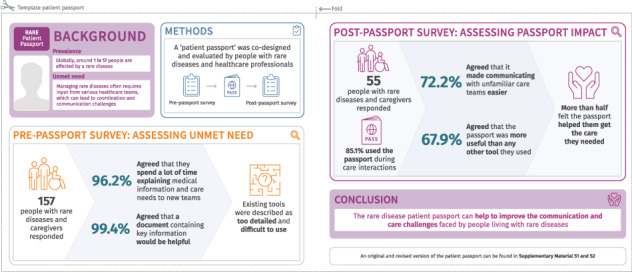

## Introduction

A rare disease is defined as affecting fewer than 1 in 2000 people within the general population [1]. Despite their individual rarity, the classification of more than 10,000 such conditions to date gives rise to an estimated 400 million people living with a rare disease globally [2–5]. As evidenced through initiatives such as the European Rare 2030 Foresight Study, there remains a need to improve awareness and coordination of healthcare for affected individuals [6].

Owing in part to the multi-systemic presentation of many rare diseases, rare disease healthcare often necessitates a multidisciplinary approach, involving healthcare professionals (HCPs) with various specialities [7–9]. However, specialists focusing on different aspects of the rare condition in isolation, exacerbated by a lack of streamlined communication between teams, often leads to challenges in accessing coordinated healthcare and positively interacting with HCPs [7]. For instance, HCPs may not be aware of, or have access to, a patient’s full medical history and needs, particularly when interacting with them for the first time. This can result in repeated tests, conflicting treatments and fragmented healthcare; such barriers and subsequent delays can negatively impact patient experiences and diminish confidence in the care they receive [10, 11].

It is also common for individuals with rare diseases to encounter HCPs who are not familiar with their condition, due to the nature of rarity but also due to limited training and the absence of rare disease specific treatment guidelines [10, 12, 13]. As a result, patients may not always receive optimal healthcare [14], and the responsibility often falls to the patient/caregiver to educate HCPs on the condition and explain their medical history. The repeated recall of this information can be especially challenging during high-stress situations such as emergency care, and such responsibility is associated with considerable psychological burden [15]. This burden may be exacerbated by individual communication preferences and abilities, which are often impacted by rare diseases [16].

‘Patient passports’ are documents that provide a summary of key information on an individual’s health and care needs, and have been developed and trialled across various medical conditions and settings [17**–**20]. These passports quickly and accurately share information about a person’s condition, healthcare team contacts, treatment plans, care preferences and emergency protocols to help HCPs understand and manage patients’ unique needs. The use of patient passports aims to improve patient experience and healthcare quality by enhancing communication with and between healthcare teams.

One such patient passport, provided to culturally diverse families of hospitalised children, was shown to aid communication with healthcare teams [17]. Another, provided to older adults being discharged from secondary care, demonstrated the additional benefit of helping patients to better manage their medications [21]. Evidence suggests patient passports facilitate consistent, patient-centred communication with HCPs and could enable better-informed clinical decisions and shared decision making [22]. In England, the NHS recently published guidance aiming to standardise healthcare passports. The guidance highlighted the benefits of passports and emphasised the importance of portability, accessibility and being customisable [22]. However, as of early 2025, there has been no consistent roll-out of patient passports across any disease area, despite ‘hospital passports’ being part of mandatory NHS training [23].

As part of NHS England’s 2018 Rare Disease Implementation Plan, it was proposed that healthcare providers issue every individual with a rare condition a ‘rare disease alert card’ [24]. This proposed tool is similar to a patient passport in how it details the individual’s disease, treatment and an expert contact, but it has yet to be implemented within the NHS [25]. A tool to consolidate medical and healthcare information could address priorities in the UK Rare Disease Strategy by improving healthcare care coordination and continuity [26]. In turn, it could support HCP decision-making and confidence in treating rare diseases. At present, there is no widely adopted, versatile patient passport that comprehensively addresses the multifaceted needs of those affected by rare diseases. The tool would need to capture the nuances of rare conditions, which may include multiple diagnoses and personalised healthcare requirements, while also detailing essential medical history to guide routine and emergency treatment plans across healthcare settings.

This pilot study assessed the utility of a patient passport for rare diseases in enhancing healthcare coordination and reciprocal patient/caregiver-care team interactions. Insights were gathered by surveying patients and caregivers with rare disease experience prior to and following use of the passport. HCPs were also invited to review the passport.

## Materials and methods

### Passport design

A patient passport and surveys to assess it were co-designed by the Cambridge Rare Disease Network (CamRARE) [27], patients with rare diseases and HCPs, the majority of whom were previously known to CamRARE through community engagement activities. The passport included sections on personal details, contact information, diagnosis, medical and emergency healthcare history and care preferences. It was made available as an editable, downloadable PDF document to be printed as a compact, hand-held resource (see Supplementary Material S1 for the passport template).

### Study design

A draft of the patient passport was initially trialled by a group of 16 children with a rare disease and their caregivers in 2022 (results not reported here) [28]. Patient and caregiver feedback was incorporated to subsequently produce the first iteration of the passport, which was used in the present pilot study, launched in 2023 (see Supplementary Fig. 1 for pilot study timelines). This pilot included individuals of all ages with a rare disease or caregivers of those with a rare disease.

Patients and caregivers were first asked to complete a survey prior to using the passport, to share their experiences of receiving care for their condition. This ‘pre-passport survey’ contained 14 questions (Supplementary Table 1). Upon completion of the survey, respondents received the patient passport to populate and use. User and reference guides were provided to participants alongside the passport to aid completion, either independently or with the help of a caregiver or family member. Patients and caregivers then received a ‘post-passport survey’, containing 21 questions (Supplementary Table 1), to evaluate the impact of the patient passport in healthcare settings. These surveys were designed in collaboration with patient representatives, comprised both multiple choice and open-ended questions and aimed to be as concise as possible. Acceptability of the length of the surveys was confirmed by feedback from patients/caregivers. Participants completed the surveys online, at their own leisure.

HCPs were presented with the patient passport alongside a 15-question survey that assessed both their general perception of patient passports and their opinion on how this passport would impact their clinical practice (Supplementary Table 2). HCP participants also completed the surveys online, at their own leisure.

### Participant recruitment

Eligible participants for the ‘pre-passport survey’ and ‘post-passport survey’ were either patients, or caregivers of a patient, with a rare disease and were recruited via rare disease networks, social media and snowball sampling, whereby existing participants freely recruited additional participants through their own social network (Supplementary Table 3).

HCPs in attendance at the Royal College of Paediatrics and Child Health (RCPCH) conference (May 2023) and the NHS East Genomics Forum for Paediatricians (July 2023) were first invited to review the patient passport and complete a separate survey; additional HCPs became involved following wider dissemination through various HCP networks, as well as through snowball sampling (Supplementary Fig. 1; Supplementary Table 3).

### Data analysis

Categorical and binary data collected via the surveys were summarised by frequencies and percentages using Microsoft Excel^®^. For quantitative data, results are presented as the proportion of total survey responses received for each question. An exploratory analysis of qualitative data, in the form of content analysis of free-text responses, was conducted. A dual coding approach was taken, whereby two individuals reviewed free-text responses and suggested recurrent themes, with a third independent reviewer arbitrating discussions until agreement for the presence of a given theme and its incident frequency was reached. No a priori coding framework was utilised. The frequency of the most notable themes identified within free-text responses are reported alongside quantitative results.

### Ethics and consent

In accordance with the verdict of the Health Research Authority decision tool, ethics committee review was not required as this study was conducted for the purpose of service evaluation and was thus not deemed research [29]. A privacy notice and participant information sheet was shared with all patient and caregiver participants; informed consent was thus gained through the act of submitting a response to the surveys.

## Results

### Pre-passport survey

By September 2023, 157 respondents had answered the pre-passport survey, 89.2% (140/157) of whom completed all questions. Of all respondents, 53.5% (84/157) were caregivers of someone living with a rare disease, and 46.5% (73/157) were living with a rare disease themselves. A total of 56 respondents disclosed their geographic location; of 55 identifiable locations, the majority were within the UK; other locations comprised Ireland, Australia, Denmark and the United States (Supplementary Table 4).

#### Experiences of interactions in care settings

**Fig. 1:**
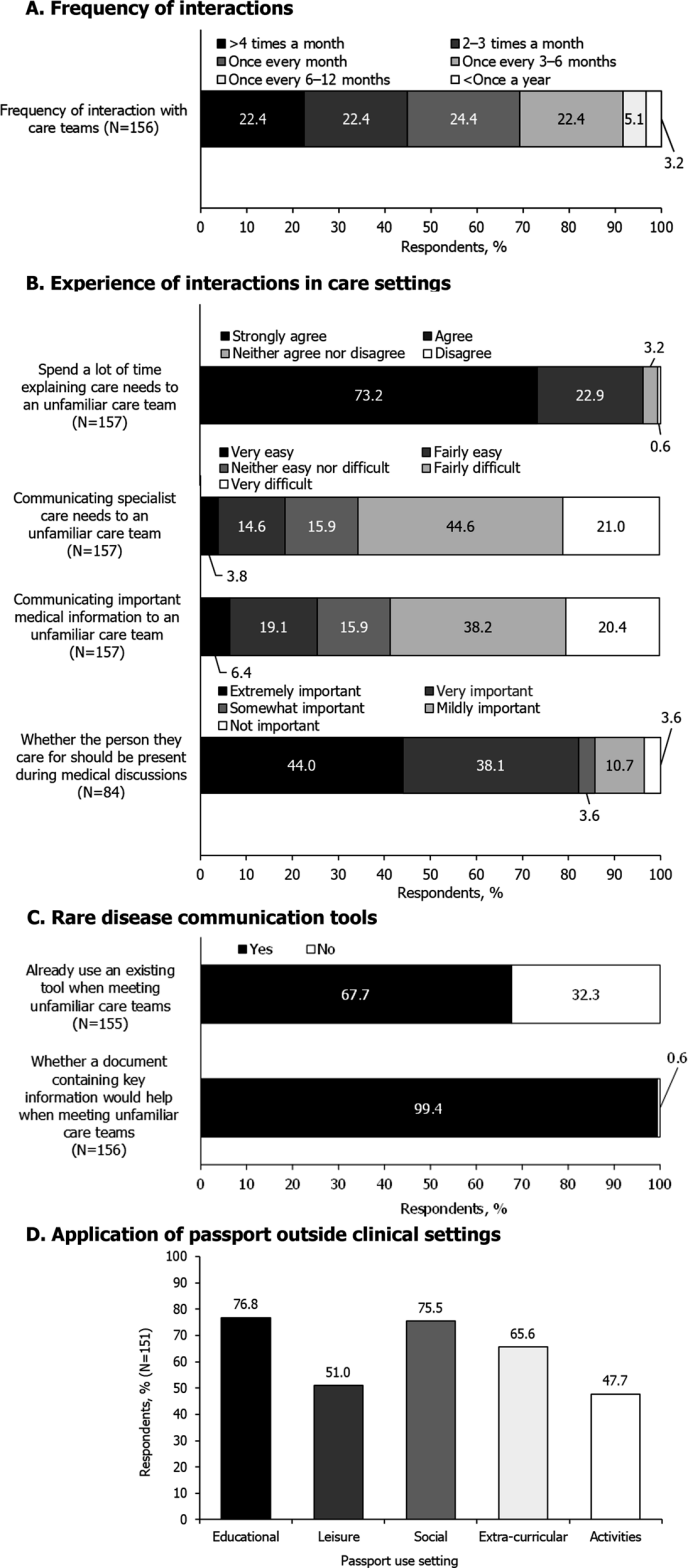
Summary of responses to the pre-passport survey. Presented data are rounded to one decimal place so may not sum to exactly 100%. **A** indicates how often survey respondents interacted with care teams. **B** demonstrates the experiences of respondents in communicating with care teams. The question on having the person they care for present during medical conversations was aimed at caregivers specifically. Strongly disagree was also an option in the question regarding time spent explaining important medical information, though was not selected by any participants. **C** indicates the respondents' experience and opinion of using a tool to aid in communication with care teams. **D** illustrates responses when participants were asked in which situations did they think a ‘hospital passport’ would be the most useful, with the option to select multiple responses. The educational setting encompassed schools; colleges and universities, leisure including restaurants; cafes; cinemas, social including with relatives; friends and/or family/babysitters, extra-curricular clubs including after-school clubs and sports clubs, and activities including soft play; bowling and swimming as examples.

The majority of respondents (69.2% [108/156]) reported that they interacted with care teams at least once a month (Fig. 1A). Respondents’ experiences of explaining medical information and communicating care needs are summarised in Fig. 1B. Regarding the main issues faced during emergency or routine care (at hospitals or other clinical settings), respondents frequently reported that HCPs possessed insufficient knowledge of their disease (emergency care settings: 103 free-text responses; routine care settings: 86 free-text responses). Eleven respondents expressed concerns that the absence of highly specialised HCP knowledge about a given rare condition may result in inappropriate treatment in emergency care. Another issue raised by respondents was the need to repeatedly explain their situation (emergency care: 14 free-text responses, routine care: 14 free-text responses). Within routine care, 12 individuals in free-text responses suggested there was insufficient communication between care teams and a lack of effective coordination across multiple care settings or disciplines. Reflecting these results, the majority of respondents (96.2% [151/157]) agreed or strongly agreed that they spend a lot of time explaining important medical information and care needs upon meeting unfamiliar care teams (Fig. 1B).

Over half of respondents (58.6% [92/157]) reported that communicating important medical information to an unfamiliar care team was fairly or very difficult (Fig. 1B). A common theme, described in free-text responses by 13 individuals, was that they found discussing their condition exhausting or distressing. Fifteen respondents in the free-text follow-up attributed their ease in relaying medical information to their extensive experience, either due to living with the condition for a long time, professional experience, or having created their own key medical information resources. A large proportion of respondents (65.6% [103/157]) reported that communicating their specialist care needs to an unfamiliar care team was fairly or very difficult (Fig. 1B). A common theme identified in the free-text follow-up (17 responses) was experiencing disbelief from HCPs or feeling unheard.

When scoring the most important topics to discuss on meeting an unfamiliar care team for the first time, the highest ranked topics were diagnosis (clinical features, additional diagnoses) and clinical information (medications, devices needed, allergies, other reasons for emergency healthcare; see Supplementary Table 5 for scoring of all topics).

#### Rare disease communication tools

When asked whether a document containing key information would help them when interacting with an unfamiliar care team, 99.4% (155/156) of respondents agreed that it would (Fig. 1C); a recurrent theme in the free-text follow-up was that such a document would help to save time (26 responses). When asked to describe what would support respondents in adapting to an unfamiliar care team, 87 free-text responses indicated the utility of a centralised summary document or information pack that provides a comprehensive description of their condition. Eleven individuals also noted a preference for a resource that is officially endorsed or accredited.

Many respondents (67.7% [105/155]) indicated that they already use a tool to streamline interactions when meeting with an unfamiliar care team (Fig. 1C). Within a free-text follow-up question, existing tools utilised included digital applications, for example applications to store notes or medical information (24 respondents), written notes (33 respondents), and compiled information documents or medical records (39 respondents). Of the 18 respondents who commented on the utility of their existing tools, 10 respondents noted the complexity of these tools, describing them as too detailed, lacking an easily accessible summary section, or being difficult to navigate. Respondents also selected settings where a patient passport would be most useful outside of a clinical environment, with educational and social settings being the options selected by the highest proportions of respondents (Fig. 1D).

### Post-passport survey

By data cut-off in October 2023, the post-passport survey had been answered by 55 respondents, 34.5% (19/55) of whom completed all questions. The average reported duration of using their patient passport was 18 weeks (See Supplementary Fig. 1 for a study timeline).

#### Use of the patient passport

**Fig. 2:**
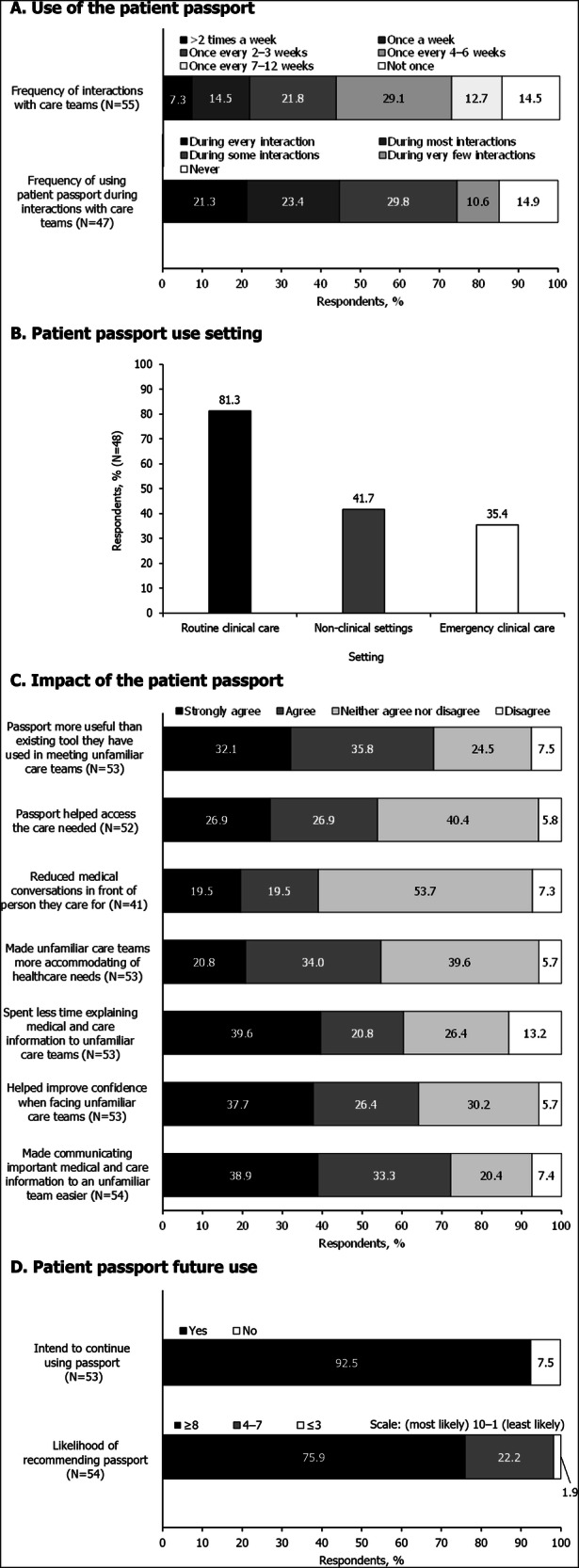
Summary of responses to the post-passport survey. See Supplementary Table 1 for full question list. Presented data are rounded to one decimal place so may not sum to exactly 100%. **A** shows the frequency with which respondents used the patient passport during the study period. **B** illustrates the settings in which the passport was used. Participants could select multiple options for their passport setting response. **C** demonstrates the respondents' perceived utility of the patient passport. Response options included ‘strongly disagree’ which was not selected by any participants. **D** shows the respondents' intentions with respect to future use or recommendation of the patient passport. Likelihood of recommendation was ranked on a scale of 1(least likely)–10 (most likely).

Since receiving the patient passport, 85.5% (47/55) of respondents reported that they had interacted with care teams (Fig. 2A), the majority of whom had used the patient passport during these interactions (85.1% [40/47]; Fig. 2A). Specifically, 44.7% (21/47) of respondents reported using the passport during every interaction or most interactions. The most common setting that the passport was used in was routine clinical care settings (81.3% [39/48]; Fig. 2B).

#### Impact of the patient passport

Responses on the impact of the patient passport, including resulting changes to interactions with care teams, are presented in Fig. 2C. A large portion of respondents (72.2% [39/54]) agreed or strongly agreed that the patient passport made communicating important medical information and specialist care needs to an unfamiliar care team easier, and over half (64.2% [34/53]) agreed or strongly agreed that the patient passport helped to improve their confidence when facing unfamiliar care teams (Fig. 2C). In the free-text follow-up, lack of HCP engagement with the passport was listed as a barrier to improved communication in nine responses. Use of the patient passport also meant that respondents had to spend less time explaining medical and care needs to unfamiliar care teams (60.4% [32/53] agreed or strongly agreed; Fig. 2C), in some cases allowing for more time to focus on the present issue in medical consultations (4 free-text responses). Only a small number of respondents (13.2% [7/53]) disagreed that less time was spent explaining their needs.

Over half of respondents either agreed or strongly agreed that the passport helped them access the care that was needed (53.8% [28/52]); however, almost half of respondents neither agreed nor disagreed with this statement (40.4% [21/52]). Over half of respondents either agreed or strongly agreed that the passport made unfamiliar care teams more accommodating of their needs (54.7% [29/53]). Furthermore, 67.9% (36/53) either agreed or strongly agreed that the patient passport was more useful than any other tool they were already using to make meeting with an unfamiliar care team easier (Fig. 2C).

#### Future use of patient passport

The majority of respondents (92.5% [49/53]) indicated they would use their passport in the future. Respondents were asked how likely they were to recommend the passport to another member of the rare disease community on a scale of 1 (least likely) to 10 (most likely), to which 75.9% (41/54) rated their likelihood as 8 or higher (Fig. 2D).

When asked about suggested future changes, a recurrent theme (reported in 12 free-text responses) centred around improving attitudes towards, or awareness of, the patient passport. Possible solutions suggested to achieve wider acceptance of the passport within medical communities included endorsement from the NHS or other healthcare organisations. Other suggestions in free-text responses included stylistic and formatting changes, such as having the ability to adapt the spacing/layout to add additional information (6 respondents), bolding the print (3 respondents) and increasing the typeface size (3 respondents).

### Healthcare professional survey

**Fig. 3:**
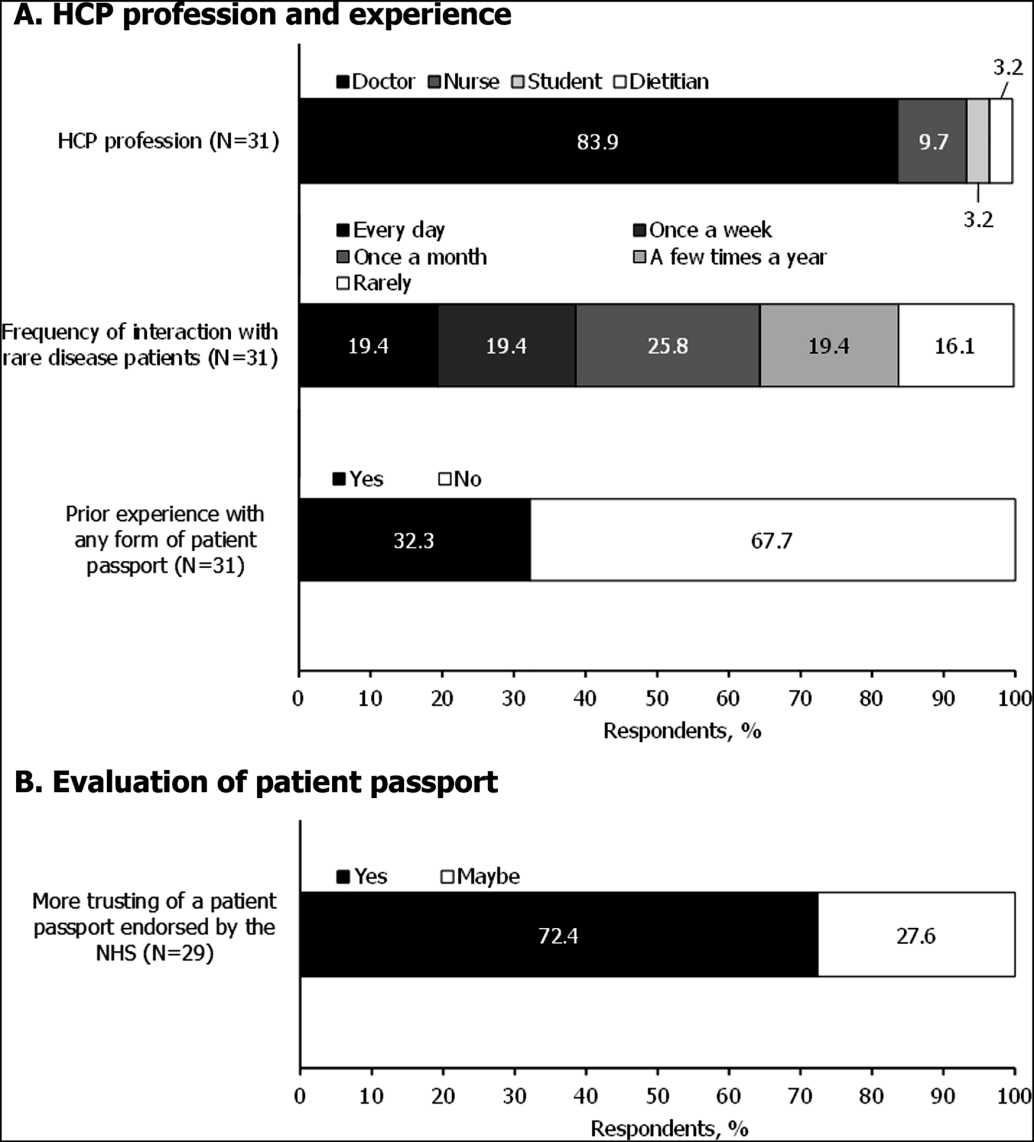
Summary of responses to the HCP survey. See Supplementary Table 2 for full question list. Presented data are rounded to one decimal place so may not sum to exactly 100%. **A** shows the HCP respondents' professions and experience with patients affected by rare disease and patient passports. **B** illustrates HCP respondents' attitudes towards official endorsement of a passport. Response options for the ‘more trusting of a patient passport endorsed by the NHS’ question included ‘Yes’, ‘Maybe’ and ‘No’.

By November 2023, the survey had been completed by 31 HCPs, the majority of whom were doctors (83.9% [26/31]) (Fig. 3A). All respondents reported having interacted with patients with rare diseases in clinical practice, 38.8% (12/31) of whom reported an interaction at least once a week. In contrast, only 16.1% (5/31) reported rarely interacting with those living with a rare disease (Fig. 3A).

Prior experience with any form of patient passport in their clinical practice was reported by 32.3% (10/31) of respondents (Fig. 3A). Of those without prior experience of a patient passport, two HCPs reported previous attempts to create a similar resource for their patients in free-text responses.

#### Evaluation of patient passport

All respondents listed perceived benefits of a patient passport for patients/caregivers via free-text responses. The most frequent benefit, expressed by 13 respondents, was improved communication between HCPs and the patient/caregiver; 4 respondents noted in addition that this would be of particular benefit in emergency/stressful settings. Other perceived benefits drawn from free-text responses included alleviating the stress associated with hospitalisation, empowering patients (4 responses each) and aiding in the development of, or helping to inform, patients’ individualised healthcare plans (2 responses).

Eighteen HCPs described potential concerns with patient-led passports in free-text responses, the most common concern being the integrity of the content, primarily whether the information is accurate and up to date (11 respondents). The absence of HCP input on the passport’s content was further raised as a concern by 4 respondents in free-text responses, as well as the risk of passports being lost/forgotten (2 respondents). When asked if they would be more trusting of a patient passport endorsed by the NHS, the majority of HCPs (72.4% [21/29]) agreed that they would (Fig. 3B). Most HCPs (78.9% [15/19]) felt data privacy concerns would not deter them from using the passport, however, 4 respondents (21.1%) did relay concerns, with apprehension surrounding data security described in 2 free-text responses.

When asked to provide additional feedback on the patient passport piloted in this study, 19 HCPs provided feedback via free-text responses. The most common theme pertained to additional information that could be captured in the passport (9 respondents), for example, details of the patients’ main healthcare team and involvement of other care teams. Four HCPs also suggested linking the passport to additional information, such as medical records or websites. Minor layout and content changes were also suggested, such as having more space for those with multiple conditions, to ensure the passport’s relevance across rare diseases (4 respondents); 2 respondents suggested to make details relevant to emergency healthcare more prominent.

## Discussion

This pilot study demonstrated that a co-designed, rare-disease-specific patient passport can help address communication and healthcare coordination challenges faced by patients with rare diseases and their caregivers. Respondents noted that existing tools/passports were inadequate, highlighting the demand for a versatile tool to efficiently convey complex healthcare needs, particularly when interacting with unfamiliar care teams. Post-passport survey results confirmed that the present passport helps to address these challenges, supporting better communication of medical information and specialist healthcare needs. Users widely agreed that the passport helped them access the care that was needed. With 3.5 million people living with a rare disease in the UK alone [30], the passport has the potential to improve care for a substantial population in the UK and beyond.

The complexity of rare diseases, which often require management by a multidisciplinary team [7–9], can result in disjointed and fragmented healthcare. This is exacerbated by the frequent lack of access to a healthcare coordinator or shared electronic records [31]. To alleviate the substantial burden placed as a result on patients and caregivers, such as having to coordinate their own healthcare, clear and consistent communication channels across healthcare teams are needed. This pilot study highlighted communication challenges between healthcare teams, with the majority of patients and caregivers reporting the need to spend considerable time explaining and repeating their medical information and care needs to unfamiliar teams. These challenges were further exacerbated by HCPs’ limited knowledge of a given rare condition, which has previously been associated with emotional distress and reduced confidence in the healthcare received [11]. The pilot study demonstrated that the consolidation of medical and care information in the form of a patient passport improved communication between patients/caregivers and HCPs, including the time spent and patients’/caregivers’ confidence in communicating. While over half of patient/caregiver participants reported that the passport helped them to access the care that was needed, almost half neither agreed nor disagreed that the passport helped them in this way. This finding suggests that although the passport holds significant potential to improve patient care, further exploration is required to identify the specific contexts where the passport is and is not best fulfilling that potential. Furthermore, the passport was shown to help care teams be more accommodating of patients’ needs and to facilitate better provision of the care required. Thus, the passport can enhance patient/caregiver experience and provides HCPs with necessary information to facilitate better-coordinated healthcare.

Despite NHS England’s recommendation to introduce an ‘alert card’ to improve rare disease healthcare [25], such a tool has yet to be consistently implemented. The present passport may address this gap by improving communication and coordination of healthcare for people living with rare diseases. Indeed, the majority of respondents found the passport more useful than any other tool they had used; this may be reflective of the passport covering topics described by respondents as the most important to discuss when first meeting an unfamiliar care team. The passport was well utilised in the pilot, possibly due to its simplicity and accessibility (being intended for use by a wide range of ages and for any rare or undiagnosed condition) relative to existing tools which were described as overly detailed and challenging to navigate by some participants. With the majority of respondents likely to recommend the passport to others in the rare disease community, findings suggest that it is well-suited to meet the broad needs of users.

An updated version of this passport (Supplementary Material S2) is now available to anyone with a rare or undiagnosed condition worldwide, with over 1,600 individuals, spanning 53 countries, having applied for the passport to date. Improvements suggested in this pilot study, such as stylistic and formatting changes, were implemented in the updated version to further enhance usability.

In the present study, a high rate of drop off was observed; of 157 participants who completed the ‘pre’ survey, 55 subsequently responded to the ‘post’ survey (i.e. ~35% of participants responded to the follow-up). This is similar, however, to the 43% survey response rate amongst recipients of a patient passport for use in a paediatric cardiology clinic, seen in another pilot study [32]. One possible reason for the high rate of drop-off is hesitancy to routinely carry the passport in its paper form. Thus, there is a need to explore digital formats, which may improve practicality and usability. To this effect, in collaboration with NHS Trusts and technology partners, digital integration of the passport into patient-facing platforms is currently being piloted, with a view to integrate the tool widely into electronic health records.

Based on the findings of this pilot study, it is hypothesised that wider dissemination and integration of this patient passport could support wide-spread improvements in communication between patients/caregivers and HCPs, and coordination of healthcare. In turn, this could lead to reduced patient and caregiver burden and improved quality of care and life for individuals with rare conditions. Testing of this hypothesis requires further investigation.

Larger-scale research is therefore warranted alongside the digital integration pilot to better evaluate the passport’s impact and usability in routine, emergency and non-clinical care settings and to validate the passport’s applicability across different geographies and healthcare systems. Such work would benefit from the application of validated patient-reported outcome measures, such as The Genomics Outcome Scale [33], which could be utilised to assess patient empowerment, aiding in evaluating the passport’s effectiveness. Frameworks like the Diffusion of Innovations Theory [34] and RE-AIM (Reach, Effectiveness, Adoption, Implementation, Maintenance) [35], could also be valuable for evaluating reach, adoption and implementation among patients and HCPs in a larger-scale roll-out of the passport. Alongside monitoring health-based metrics and translation of surveys into multiple languages, integrating these validated tools and frameworks could help to reduce the positive skew and bias associated with a small pilot study. With the majority of HCPs indicating the desirability of official endorsement (for example, from the Department of Health and Social Care in the UK), it is hoped, in turn, that such research would support this aim and further enhance trust in the passport.

### Strengths and limitations

#### Patient/caregiver surveys

This study included various means of participant recruitment, which provided valuable real-world insights into the lives of the diverse rare disease community and their interactions in different healthcare settings. Key limitations included a reduction in response rate between pre-passport and post-passport surveys. This may have introduced bias toward those who favoured the passport, as continued engagement was likely associated with favourable opinions. Similarly, while the majority of post-survey respondents (85.5%) reported that they had interacted with care teams since receiving the passport, reported data included those who had not; the absence of such experience may have influenced their interpretation of its usefulness. The absence of sociodemographic data collection in the surveys limited the ability to report on the representativeness of participants, thus restricting assessment of the wider applicability of the findings.

#### HCP survey

The small sample size of 31 respondents to the HCP survey and the distribution of the survey amongst individuals within CamRARE’s network may have introduced bias due to organisational familiarity. Despite varying professions (among those who voluntarily chose to report it), the sample doubtless lacked comprehensive coverage of the myriad of professions that can be involved in patient care for rare diseases. Finally, HCP participants reviewed a copy of the patient passport without direct experience of using it in clinical practice, meaning their feedback was limited to perceived usefulness. Future research would benefit from the involvement of HCPs with direct experience of using the passport in clinical settings to better assess its impact.

#### Qualitative analysis

While the qualitative summary of free-text responses into key themes enabled a deeper exploration of respondents’ unmet needs and their evaluation of the passport, the pilot study was designed to evaluate quantitative responses, with free-text responses in the surveys providing opportunity for additional context. Since no coding framework was developed to guide analysis, a level of subjective interpretation was required. Thus, while free-text responses were generally short and required minimal interpretation, qualitative findings must be interpreted with appropriate caution.

## Conclusion

The results of this pilot study highlight the potential impact of this rare disease-specific patient passport, co-designed with patients, to improve communication between patients/caregivers and HCPs, and coordination of healthcare for individuals with rare diseases. By streamlining interactions across various healthcare settings, findings suggested that the passport helped to reduce the burden of responsibility on patients and caregivers. Healthcare teams were reported to be more accommodating of patients’ needs, helping them access the care they needed, which may ultimately improve patient experience and outcomes. The passport was regularly used by participants in this pilot study, considered by most as more useful than any other tool they had used, and many respondents would recommend the passport to their peers. Together, these findings strongly support the passport’s integration into healthcare systems. However, further work is warranted to explore its usability and effectiveness across diverse healthcare settings, enhance HCP engagement through seeking formal endorsement, and to identify the contexts in which it would be most useful. Such efforts would maximise access to and uptake of the passport, ultimately improving patient care and experience.

## Supplementary information

Patient Passports for Rare Diseases – Supplementary Material

## Data Availability

The datasets generated during and/or analysed during the current study are available from Costello Medical on reasonable request.
