# Supplementary material for: Patient passports for rare diseases: results of a pilot study
Source: Eur J Hum Genet. 2025 Oct 14;34(1):99–107. doi: 10.1038/s41431-025-01930-w (PMC12816684; doi:10.1038/s41431-025-01930-w)
Supplement: Supplementary file 1 — Patient Passports for Rare Diseases – Supplementary Material [file 41431_2025_1930_MOESM1_ESM.pdf]

SUPPLEMENTARY MATERIAL

Supplementary Figure 1. Service evaluation pilot study timelines

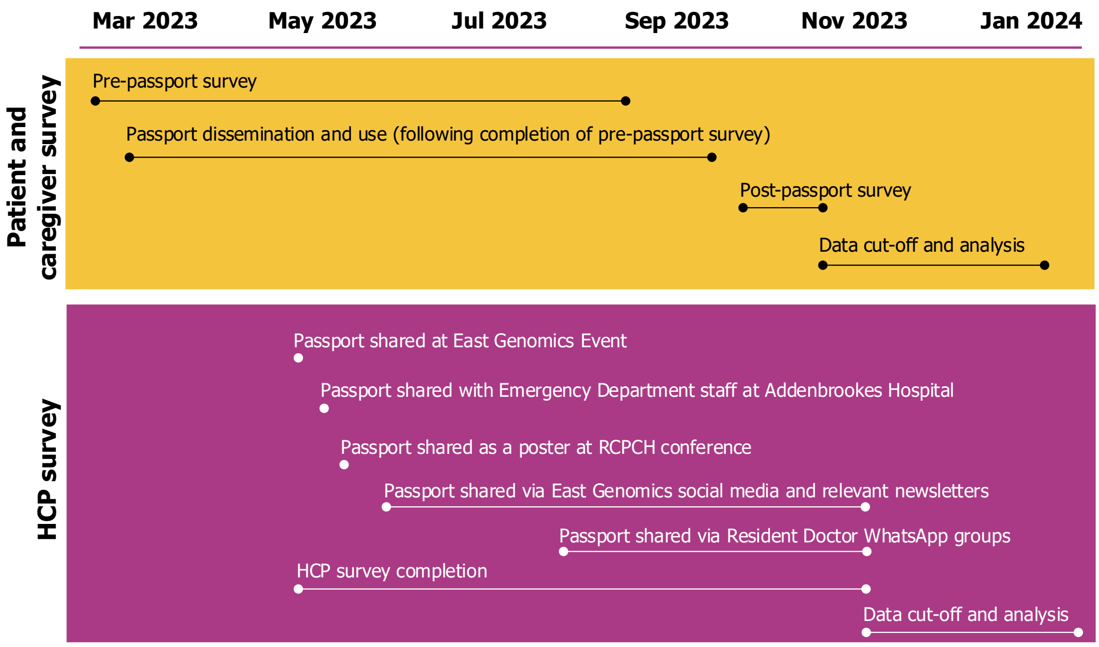

Participants received access to the passport immediately following completion of the pre-passport survey. The range for time using the passport was 4–53 weeks, though the respondent who answered 53 weeks was identified as a participant in the initial 2022 trial who had incorrectly included this period in their response. Abbreviations: CamRARE, Cambridge Rare Disease Network; HCP, Healthcare Practitioner; RCPCH, Royal College of Paediatrics and Child Health.

Supplementary Material S1. The Rare Patient Passport template.

THIS IS ME

Rare Disease Passport

ADD PHOTO HERE
25MM X 30MM

My name is >
I like to be known as >
Date of birth > / /
NHS no. >
Travel insurance no. >

Completed > / / by >

MY DIAGNOSIS >>

I am diagnosed with >

Key clinical features > > > > >

Brief description >

KEY CONTACTS >>

MEDICAL CONTACTS:

Specialist's name >
Clinic >
Hospital >
Phone no. >
Specialist's name >
Clinic >
Hospital >
Phone no. >

I am also diagnosed with >

Key clinical features > > > > >

Brief description >

GP's name >
GP surgery address >
Phone no. >

EMERGENCY CONTACTS:

1st emergency contact >
Relationship > Phone no. >
Email >

2nd emergency contact >
Relationship > Phone no. >
Email >

KEY CLINICAL INFO >>

Medications > > > >

Devices needed > > >

Allergies >

3 TOP THINGS TO KNOW ABOUT ME >>

1 >

2 >

3 >

Please present my This Is Me passport to medical staff on my behalf

Respiratory issues >

Seizures >

Common reasons why I may require emergency care >

MORE OVERLEAF >>

A&E/CRITICIAL CARE RECORD >>

How frequently I require emergency care (on average): weekly / monthly / annually / less often

HISTORICAL INSTANCES OF CRITICAL CARE

Date	Presentation	Treatment	Outcome
> / /	>	>	>
> / /	>	>	>
> / /	>	>	>

MY NORMAL >>

Height >
O_2 sats >
Weight >
BP >
Pain >
Neurology >
Major surgery >

CARING FOR ME >>

In a hospital, it helps me if >
When you speak to me it helps if >
When I am tested, I prefer >

Implants >
Lines / tubes >
Communication >
Mobility >

Eating & drinking >
Things I enjoy >
Things I don't like >

Vision >
Hearing >
Toileting >
Equipment I use >

Other things about me >

Designed by camRARE Cambridge Rare Disease Network

Supplementary Material S2. The Rare Patient Passport template (updated as of April 2024)

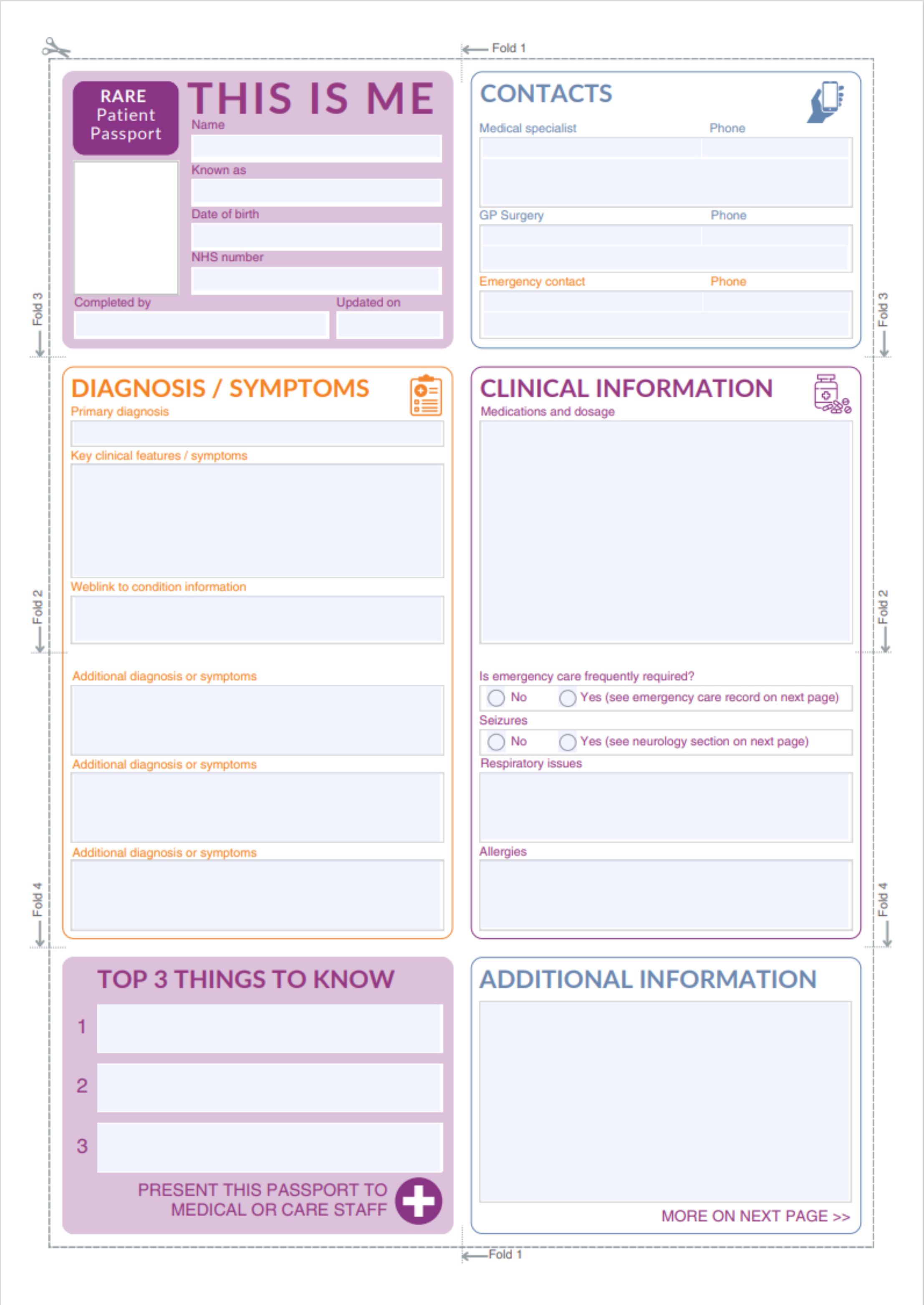
Fold 1

RARE Patient Passport

THIS IS ME

Name

Known as

Date of birth

NHS number

Completed by

Updated on

CONTACTS

Medical specialist | Phone

GP Surgery | Phone

Emergency contact | Phone

Fold 3

DIAGNOSIS / SYMPTOMS

Primary diagnosis

Key clinical features / symptoms

Weblink to condition information

Additional diagnosis or symptoms

Additional diagnosis or symptoms

Additional diagnosis or symptoms

CLINICAL INFORMATION

Medications and dosage

Is emergency care frequently required?

No | Yes (see emergency care record on next page)

Seizures

No | Yes (see neurology section on next page)

Respiratory issues

Allergies

Fold 2

Fold 4

TOP 3 THINGS TO KNOW

1

2

3

PRESENT THIS PASSPORT TO MEDICAL OR CARE STAFF

ADDITIONAL INFORMATION

MORE ON NEXT PAGE >>

Fold 1

Fold 1

EMERGENCY CARE RECORD
HISTORICAL INSTANCES OF A&E VISITS

Average frequency of emergency care
◯ weekly ◯ monthly ◯ annually ◯ less often

Date	Presentation / symptoms	Treatment	Outcome

Fold 3

MY NORMAL

Height

Weight

Pain

Neurology & neurodivergence

Major surgery history

Implants / lines / tubes

Mobility

Sensory impairments

Equipment & devices used

Toileting

Additional information about me or about caring for me

CARING FOR ME

Key things to know about caring for me

Communication

Eating & drinking

Likes

Dislikes

Fold 2

Fold 4

This passport template was designed by

camRARE

www.camraredisease.org

DISCLAIMER

This document reflects the patient or caregiver's understanding of their condition and is to be used as a tool to aid communication of medical and care needs. CamRARE is not liable for any losses or damages from the use of this passport or its information.

Fold 1

Supplementary Table 1: Pre-passport and post-passport survey questions

Theme	Question	Format
**Pre-passport survey questions**		
**Care team interaction**	How often do you interact with care teams?<br><br>Options:<br>• >4 times a month<br>• 2–3 times a month<br>• Once a month<br>• Once every 3–6 months<br>• Once every 6–12 months<br>• Less than once every year	Categorical
**Issues faced in clinical settings**	Please complete the following sentence:<br><br>When I or the person I care for enter hospitals or other clinical settings for emergency care, the main issues I face are...	Free text
	Please complete the following sentence:<br><br>When I or the person I care for enter hospitals or other clinical settings for routine care, the main issues I face are...	
**Communicating disease information**	†How do you find communicating important medical and health (or clinical) information about you or the person that you care for to an unfamiliar care team?<br><br>Options:<br>• I find it very easy<br>• I find it fairly easy<br>• I find it neither easy nor difficult	Categorical

Theme	Question	Format
	• I find it fairly difficult<br>• I find it very difficult	
	When you are faced with an unfamiliar care team, how do you feel about communicating the specialist care needs that you or the person you care for may require?<br><br>Options:<br>• I find it very easy<br>• I find it fairly easy<br>• I find it neither easy nor difficult<br>• I find it fairly difficult<br>• I find it very difficult	Categorical
	Follow-up: If you find it difficult, please can you share any thoughts about what makes it difficult?	Free text
	*†Please rate how much you agree with the following statement:<br><br>I find I spend a lot of time explaining important information about the medical and care needs of myself/the person I care for upon meeting unfamiliar care teams for the first time.	Categorical
**Reasonable adjustments within healthcare systems**	Please complete the following sentence:<br><br>Facing/adapting to an unfamiliar care team would be easier for me and/or the person I care for if…	Free text
	*†If you are a caregiver, how important is it to you to be asked whether the person you are caring for should be present while having medical conversations?	Categorical

Theme	Question	Format
**Rare disease communication tools**	Is there any tool you use to make meeting with an unfamiliar care team easier? e.g. taking notes into the meeting, another passport or ID card, an app on your phone where you store information about health and care needs. (Yes/No)	Free text
	Follow-up: [If yes] Please explain what the tool is. How does this help and is there anything that does not work well about it?	Binary
	Would a document with key information relating to the person with a rare disease make meeting an unfamiliar care team easier? (Yes/No)<br><br>Follow-up: [If yes] Why?	Binary<br><br>Free text
	Outside of clinical settings, in what situations do you think a hospital passport would be the most useful? Please select all that apply.<br><br>Options:<br>• Educational (schools, college, university)<br>• Extra-curricular clubs (after-school clubs, sports clubs, other clubs)<br>• Leisure (restaurants, cafes, cinemas)<br>• Activities (soft play, bowling, swimming)<br>• Social/personal/home (with relatives, friends and/or family, babysitters)	Categorical
	As a person with a rare condition or a caregiver, what are the most important topics to discuss when meeting an unfamiliar care team for the first time?<br><br>(see **Supplementary Table 5** for categories)	Categorical
	If you or the person you care for could rename the hospital passport document, what would you change it to? Please feel free to make you own suggestions using the 'Other' option.	Categorical

Theme	Question	Format
	Options:<br>• Keep it as 'Hospital Passport'<br>• 'This Is Me' document<br>• CareCard<br>• My Care Guide<br>• Other<br><br>Follow-up: [If other] Please suggest a name for the care document	 <br><br><br><br>Free text
**Post-passport survey questions** *(Note that the patient passport is described as the 'This is Me' rare disease passport within the relevant questions here)*		
**Participation**	Did you take part in the 'CamRARE Rare Patient Passport Pre-Phase Two Survey'? (Yes/No)	Binary
**Passport use**	How long have you been using your 'This is Me' rare disease passport? Please answer in number of weeks, e.g. '6 weeks'	Free text
	How often have you interacted with care teams since you started using your 'This is Me' rare disease passport?<br><br>Options:<br>• >2 times a week<br>• Once a week<br>• Once every 2–3 weeks<br>• Once every 4–6 weeks<br>• Once every 7–12 weeks<br>• Not once	Categorical

Theme	Question	Format
**Passport setting use**	How often did you use your 'This is Me' rare disease passport during these interactions [with care teams]?<br><br>Options:<br>• During most interactions<br>• During some interactions<br>• During very few interactions<br>• Never	Categorical
	What settings did you use your 'This is Me' rare disease passport in? (Please select all that apply)<br><br>Options:<br>• Emergency clinical care<br>• Routine clinical care<br>• Non-clinical settings	Categorical
	If you used your 'This is Me' rare disease passport in a non-clinical setting, please select the relevant settings (Please select all that apply)<br><br>Options:<br>• Educational (schools, college, university)<br>• Extra-curricular clubs (after-school clubs, sports clubs, other clubs)<br>• Leisure (restaurants, cafes, cinemas)<br>• Activities (soft play, bowling, swimming)<br>• Social/personal/home (with relatives, friends and/or family, babysitters)	Categorical
	Please complete the following sentence:	Free text

Theme	Question	Format
**Issues addressed by passport**	The Main issue the 'This is Me' rare disease passport helped with when I or the person I care for entered a hospital or other clinical setting for emergency care was...	
	Please complete the following sentence:<br><br>The Main issue the 'This is Me' rare disease passport helped with when I or the person I care for entered a hospital or other clinical setting for routine care was...	
	Please complete the following sentence:<br><br>The main issue the 'This is Me' rare disease passport helped me or the person I care for with in non-clinical settings was...	
	*†The 'This is Me' rare disease passport made communicating important medical information and specialist care needs about me or the person that I care for to an unfamiliar care team easier	Categorical
	*†By using the 'This is Me' rare disease passport, I found that I had to spend less time explaining important information about the medical and care needs of myself or the person I care for upon meeting unfamiliar care teams for the first time	Categorical
	*†The 'This is Me' rare disease passport made me feel more confident when facing an unfamiliar care team	Categorical
	*†The 'This is Me' made unfamiliar care teams more accommodating of my needs or those of the person I care for	Categorical
	*†The 'This is Me' helped me or the person I care for get the care I/they need	Categorical
	*†As a caregiver, the 'This is Me' rare disease passport reduced the number of times I had to have medical conversations in front of the person I care for	Categorical

Theme	Question	Format
**Passport advocacy**	*†The ‘This is Me’ rare disease passport was more useful than any other tool I was already using (e.g. notes, another passport or ID card, medical letters) in making meeting unfamiliar care teams easier	Categorical
	Are you planning on using the ‘This is Me’ rare disease passport in the future? (Yes/No)<br><br>Follow-up 1: If ‘Yes’, what settings are you planning on using the hospital passport in? (Please select all that apply)<br><br>Options:<br>• Emergency clinical care<br>• Routine clinical care<br>• Non-clinical settings<br><br>If 'No', can you tell us why?	Binary<br><br>Categorical<br><br><br><br>Free text
	How likely would you recommend the ‘This is Me’ rare disease passport to another member of the rare disease community? (1 = very unlikely, 10 = very likely)	Categorical
**Passport suitability**	Please complete the following sentence:<br><br>The most useful feature of the 'This is Me’ rare disease passport is...	Free text
	If there was one thing you could change about the ‘This is Me’ rare disease passport, what would it be?	Free text
**N/A**	Please provide any other comments or feedback you have on the ‘This is Me’ rare disease passport below	Free text

For the categorical questions marked with an asterisk (*), the options for respondents to choose from were as follows: Agree, Strongly agree, Neither agree nor disagree, Disagree, Strongly Disagree. Those marked with † also had an associated free-text response question in the format: ‘Please feel free to share any comments to expand on your selected answer to Question X’.

Supplementary Table 2: HCP survey questions

Theme	Question	Format
**Care role**	What is your role? Please specify (eg. Doctor, Nurse, Social Worker)	Free text
**Location**	Where are you based in the UK?	Free text
**Patient passport prior experience**	Have you been presented with any patient passport in your practice? If yes, please describe your experience.	Free text
**Patient passport benefits**	What benefits do you think the patient passport provides for the patient/caregiver?	Free text
	What benefits do you think the patient passport provides for the care provider?	Free text
**Patient passport content**	What information you would like to see for a patient that has a rare or undiagnosed disease?	Free text
**Rare disease patient experience**	Are you aware of having treated a patient with a rare disease? If yes, how often do you see rare disease patients in your practice? [Answering the question constitutes as a 'Yes' response]<br><br>Options:<br>• Every day<br>• Once a week<br>• Once a month<br>• A few times a year<br>• Rarely	Categorical
	Have you experienced any challenges in treating rare disease patients?	Free text
**Patient passport concerns**	Do you have concerns about the risks of a patient-led passport? If yes, what are they?	Free text
	Do data privacy concerns deter you from using the passport? If yes, can you please expand?	Free text
**Credibility of the patient passport**	When patients present information to you, what makes it credible?	Free text

Theme	Question	Format
	If a passport was supported by the NHS, would you be more trusting of it?	Categorical
**Patient passport feedback**	Can you please review and evaluate the current CamRARE passport? Please provide your comments and suggestions, if any.	Free text
	What passport format would you prefer to work with?<br><br>Options:<br>• PDF print-out<br>• QR code that links to electronic PDF<br>• App on a phone	Categorical
	How often should the passport be updated and by whom?	Free text

Supplementary Table 3: Network and social media channels utilised for participant recruitment

	Patient and Caregiver Recruitment	HCP Recruitment
**Networks**		
Metabolic Support UK	✓	
Beacon	✓	
Rare Revolution	✓	
Rare Revolution Magazine	✓	
NHS East Genomics	✓	
Liverpool Centre For Genomic Medicine	✓	
Alder Hey Children’s Hospital Trust	✓	
Alex – The Leukodystrophy Charity	✓	
Behçet’s UK	✓	
Fibrodysplasia Ossificans Progressiva Friends (FOP Friends)	✓	
22q11 Ireland	✓	
Pregnancy Associated Osteoporosis	✓	
Genetic Overgrowth PI3K Support (GOPI3KS)	✓	
Pitt Hopkins UK	✓	
**Social Media Channels**		
X (previously twitter)	✓	
Facebook	✓	
Instagram	✓	
LinkedIn	✓	
WhatsApp		✓
**Congress Attendees**		
Royal College of Paediatrics and Child Health (RCPCH) Conference (2023)		✓
NHS East Genomics Forum for Paediatricians (2023)		✓
**Other**		
Emergency Department staff at Addenbrooke’s Hospital		✓
East Genomics social media and relevant newsletters		✓

Supplementary Table 4: Respondent geographical demographics

Country, N (%)	Region	Proportion of patients, n (%)
**UK** 49 (89.1%)	East of England	15 (27.3%)
	West Midlands	7 (12.7%)
	South East England	6 (11.0%)
	South West England	5 (9.1%)
	Scotland	3 (5.5%)
	Wales	1 (1.8%)
	Northern Ireland	3 (5.5%)
	North West England	3 (5.5%)
	North East England	2 (3.6%)
	London	2 (3.6%)
	East Midlands	1 (1.8%)
	Yorkshire and Humber	1 (1.8%)
**Australia** 3 (5.6%)	New South Wales	2 (3.6%)
	Queensland	1 (1.8%)
**Denmark** 1 (1.8%)	Capital Region	1 (1.8%)
**Ireland** 1 (1.8%)	Eastern and Midland	1 (1.8%)
**United States** 1 (1.8%)	Maryland	1 (1.8%)

A total of 55 respondents provided identifiable details of their location; 1 other response was not able to be definitively assigned a region due a lack of geographic specificity.

Supplementary Table 5: Rating of importance of topics when meeting unfamiliar care teams

Category	n	Range	Average
Diagnosis (clinical features, additional diagnoses)	157	1–10	9.4
Clinical information (medications, devices needed, allergies, other reasons for emergency care)	157	2–10	9.2
Care preferences (eating and drinking, personal care needs, preferences for tests, communication preferences)	157	1–10	7.8
Key contacts (specialist, GP and emergency contacts)	156	1–10	7.7
A&E critical care record	157	1–10	7.3
Identity (name, date of birth, email address, NHS no.)	156	1–10	6.7
Normal measurements/traits (height, weight, communicative ability, mobility, toileting)	156	1–10	6.6

Categories and responses for the pre-passport survey question: 'As a person with a rare condition or a caregiver, what are the most important topics to discuss when meeting an unfamiliar care team for the first time?'. Participants were asked to rate each option on a scale of increasing importance from 1–10.
